# The Generation of a Testicular Peritubular Cell Line from Giant Pandas (*Ailuropoda melanoleuca*)

**DOI:** 10.3390/cells14181426

**Published:** 2025-09-11

**Authors:** Xueni You, Yuren Wang, Yuliang Liu, Rong Hou, Yi Zheng, Junhui An

**Affiliations:** 1 The Conservation of Endangered Wildlife Key Laboratory of Sichuan Province, Chengdu 610081, China; 2 Key Laboratory for Animal Genetics, Breeding and Reproduction of Shaanxi Province, College of Animal Science and Technology, Northwest A&F University, Yangling 712100, China; 3 Chengdu Research Base of Giant Panda Breeding, Chengdu 610081, China

**Keywords:** testicular peritubular cell, testis, giant panda, immortalization, SV40 large T antigen

## Abstract

Giant pandas (*Ailuropoda melanoleuca*), a flagship endangered species under priority preservation in China, remain poorly understood in terms of their testicular physiology and the mechanisms underlying spermatogenesis. Testicular peritubular cells (TPTCs), a crucial somatic cell type surrounding seminiferous tubules, secrete growth factors such as GDNF and CSF1 and release inflammatory factors such as IL-6 and IL-1β, contributing to the testicular niche and immune homeostasis. The contraction of TPTCs also facilitates the transport of sperm towards the epididymis. Nonetheless, TPTCs tend to undergo replicative senescence in vitro, which is a hinderance to their in-depth study. Here, we generated an immortalized monoclonal cell line with TPTC identities from giant pandas via lentiviral transduction of SV40 large T antigen into the cells and the subsequent clonal isolation through limiting dilution. The generated cell line, designated PD-TPTCs, demonstrated unlimited proliferative capacity and has been cultured for over five months and passaged more than 50 times to date. Characterization of PD-TPTCs revealed stable expression of key TPTC markers including ACTA2, MYH11, CNN1, and AR. Moreover, PD-TPTCs could respond to ATP and forskolin (FSK) stimulation with a pro-inflammatory gene expression profile and increased steroidogenic activity, respectively, and they were also amenable to lipofection. As such, the generated PD-TPTC line represents a promising cellular model for future mechanistic studies on the testicular niche, spermatogenesis, and reproductive disorders in giant pandas, laying the foundation for the development of novel assisted reproductive technology (ART) in this endangered species.

## 1. Introduction

Spermatogenesis, the process by which mature sperm are produced, is an extraordinarily precise and intricate biological event that safeguards the transmission of genetic information to the next generation. Spermatogenesis occurs in the testis, the male gonad where sex hormones are also synthesized, and maintenance of the testis’ function relies on intricate interactions between germ cells and various types of somatic cells. In addition to the well-characterized Sertoli and Leydig cells, testicular peritubular cells (TPTCs) have increasingly been recognized in recent years as a major somatic cell population that plays indispensable roles in testis function [1].

TPTCs form the outermost layer surrounding the seminiferous tubules. They exhibit contractile properties, facilitating the transport of sperm towards the epididymis, and display typical smooth muscle-like features, with expression of characteristic markers such as α-smooth muscle actin (ACTA2) and calponin (CNN1) [2,3]. TPTCs also possess both endocrine and paracrine functions. For instance, they secrete cytokines such as glial cell line-derived neurotrophic factor (GDNF) and colony-stimulating factor 1 (CSF1) that are important to the self-renewal of spermatogonial stem cells (SSCs) [4,5], and they produce a variety of inflammatory factors such as IL-6 and IL-1β [6], contributing to the testicular niche and immune homeostasis [7]. Under pathological conditions such as male infertility or testicular fibrosis, TPTCs often undergo morphological alterations, along with changes in cytokine expression profiles and increased extracellular matrix (ECM) deposition, suggesting that TPTCs may play intricate regulatory roles in testicular development and diseases that remain incompletely understood [8].

To gain more knowledge about TPTCs, both primary and immortalized cell models have been established in mammalian species. For instance, Albrecht and colleagues isolated and cultured TPTCs from human testis tissue [9], whereas Schmid and coauthors introduced human telomerase reverse transcriptase (hTERT) into TPTCs from the common marmoset (*Callithrix jacchus*), generating an immortalized monkey TPTC line with stable morphology and preserved functional properties [10]. These cells have been demonstrated to contribute to mechanistic studies and drug screening, and extensive data obtained from these in vitro models have disclosed numerous TPTC-related features such as marker expression, cytokine secretion profiles, contractile response, and drug sensitivity. An illustration of this point is that human TPTCs maintained in vitro express classical smooth muscle markers, such as ACTA2 and CNN1 [11], and exhibit a robust response to stimuli such as ATP and forskolin (FSK) [10,12,13], indicative of their physiological plasticity and relevance.

Giant pandas (*Ailuropoda melanoleuca*), a flagship endangered species under priority preservation in China, remain poorly understood in terms of their reproductive biology. One of the underlying reasons is their inherently low reproductive capacity, and because of this, arduous efforts have been made to enhance reproduction in this species. In addition to traditional assisted reproductive technology (ART), some novel approaches such as interspecies pregnancy, i.e., implantation of cloned giant panda embryos into the uterus of a surrogate from a different species, have been attempted, which resulted in fetal development but without live birth [14]. In a recent study, primary fibroblasts were isolated from giant pandas and reprogrammed into induced pluripotent stem cells (GPiPSCs) using non-integrative episomal vectors [15], paving the way for in vitro gametogenesis in this endangered species. Despite all these, due to the paucity of samples, the cellular composition of the testes and the mechanisms underlying spermatogenesis remain poorly understood in giant pandas. Recently we have, for the first time to our knowledge, conducted a 10 × genomics-based single-cell RNA-sequencing analysis on an adult giant panda testis, and annotated and characterized the giant panda testicular somatic cell populations including TPTCs at the molecular level [16]. We also characterized the giant panda TPTCs in situ by conducting ACTA2 immunostaining on the testis’ sections, revealing one single layer of TPTCs surrounding the seminiferous tubules in giant pandas and ACTA2 as a conserved marker for this cell type in mammalian species [16]. Another study, employing label-free quantitative proteomics, revealed stage-specific protein expression patterns in giant panda testes at distinct developmental stages and identified biomarkers related to reproductive development [17]. These findings provide novel insights into the reproductive physiology of this endangered species.

Cell immortalization enables cells to proliferate unlimitedly in vitro while retaining the original cellular characteristics, thereby facilitating the relevant functional and mechanistic studies. Concerning TPTCs, they tend to undergo replicative senescence in vitro [5], which is a hinderance to their in-depth studies, especially in non-model species like giant pandas. To obtain a stable and abundant cellular resource for giant panda TPTCs, thereby bettering the biological understanding of this peculiar cell type, we, for the first time, generated an immortalized monoclonal cell line with TPTC identities from giant pandas. The generated cell line not only represents a promising cellular model to investigate the mechanisms underlying the testicular niche, spermatogenesis, and reproductive disorders of giant pandas, but also lays the foundation for the development of novel ART and preservation strategies in this endangered species.

## 2. Materials and Methods

### 2.1. Animals

Testis samples were obtained from an adult giant panda raised in Chengdu Research Base of Giant Panda Breeding, Chengdu, China. Specifically, the donor was an 18-year-old male giant panda named Fufu. In January 2019, during a routine physical examination, it was noticed that its left testis was enlarged and had a firmer texture. The subsequent ultrasound examination revealed hypoechoic and hyperechoic masses in the left testis, leading to the preliminary diagnosis of tumors in the left testis. To circumvent metastasis, orchidectomy was performed for the left testis in February 2019. Later, pathological changes were also observed in its right testis, followed by orchidectomy for the right testis in May 2019. Upon removal of the right testis, the tumorous tissue was carefully separated, and only the normal healthy tissue was utilized for a battery of subsequent experiments. Specifically, a small part was used for fixation and staining, and the remainder was all used for enzymatic dissociation. All animal procedures abided with and were approved by the institutional animal ethical committee of Northwest A&F University (Approval Code: DK-20240422; Approval date: 21st March 2024) and the affiliated regulation enforcement agency in Chengdu Research Base of Giant Panda Breeding.

### 2.2. Hematoxylin and Eosin (H&E) Staining and ACTA2 Immunostaining

H&E staining and ACTA2 immunostaining were carried out as previously depicted [16]. In brief, the normal healthy tissue from the right testis of the donor was cut into small pieces, followed by fixation with diluted Bouin’s solution and embedding in paraffin. After slicing at a thickness of 5 μm, testis sections were deparaffinized, rehydrated, and stained with H&E. For ACTA2 immunostaining, testis sections were subjected to heat-mediated antigen retrieval in the sodium citrate buffer. After blocking with the blocking buffer (37580, Thermo Fisher Scientific, Waltham, MA, USA) for 2 h at room temperature, testis sections were incubated with mouse anti-ACTA2 (1:50; BM0002, Boster Biological Technology, Pleasanton, CA, USA) primary antibody at 4 °C overnight. For negative controls, the primary antibody was replaced with the isotype mouse immunoglobulin G. After washing on the following day, testis sections were incubated with goat anti-mouse secondary antibody conjugated to HRP (1:50; A0216, Beyotime Biotechnology, Shanghai, China) for 1 h at room temperature. Then, testis sections were stained with DAB (P0202, Beyotime Biotechnology, Shanghai, China) and counterstained with hematoxylin, followed by visualization and image capture under a microscope.

### 2.3. Isolation of TPTCs

Giant panda testicular single-cell suspension was obtained following previously reported methodology [16]. Briefly, after removal of the tunica albuginea and the surrounding connective tissue, the normal healthy tissue from the right testis of the donor was minced into small fragments and exposed to Dulbecco’s Modified Eagle Medium (DMEM, high glucose; 10-013-CVRC, Corning, New York, NY, USA) containing 2 mg/mL collagenase type IV (17104019, Thermo Fisher Scientific, Waltham, MA, USA) at 37 °C for 20 min with gentle shaking, followed by centrifugation at 80× *g* to remove interstitial cells and erythrocytes, and the precipitate containing seminiferous tubule fragments was collected and incubated with 0.25% trypsin-EDTA (Hyclone, Logan, UT, USA) and 0.5 mg/mL DNase I (Thermo Fisher Scientific, Waltham, MA, USA) at 37 °C for 5 min. Then, fetal bovine serum (FBS; FBS-CE500, Newzerum, Christchurch, New Zealand) was added to terminate the enzymatic dissociation, followed by filtration through a 40 μm mesh. After centrifugation at 450× *g* for 5 min, cell pellets containing TPTCs were aliquoted and cryopreserved for future use.

### 2.4. Cell Culture

An aliquot of testicular somatic cells was thawed and subjected to the standard culture. Both the primary and immortalized testicular cells including PD-TPTCs were cultured with a complete medium consisting of DMEM (high glucose; 10-013-CVRC, Corning, Corning, NY, USA), 5% FBS (FBS-CE500, Newzerum, Christchurch, New Zealand), 1 × GlutaMAX (35050061, Thermo Fisher Scientific, Waltham, MA, USA), 1 × non-essential amino acids (NEAA; 11140050, Thermo Fisher Scientific, Waltham, MA, USA), 1 × penicillin-streptomycin (15140122, Thermo Fisher Scientific, Waltham, MA, USA), and 1 × mycoplasma inhibitor (40607ES08, Yeasen Biotechnology, Shanghai, China). Cells were maintained at 37 °C in an atmosphere of 5% CO_2_ in air, refreshed every two days, and passaged at a ratio of 1: 3 when arriving at around 80% confluency.

### 2.5. Immortalization of Giant Panda Testicular Cells

Cell immortalization was conducted as previously depicted [18]. Briefly, the plox-Ttag-iresTK transfer vector (Addgene No. 12246) coding for the SV40 large T antigen, as well as the second-generation packaging vectors psPAX2 and pMD2.G, were co-transfected into HEK293T cells to generate lentiviral particles. Lentiviruses were then collected, ultracentrifuged for concentration, and titrated. For cell immortalization, the primary testicular somatic cells at passage 3 were seeded into 24-well plates for lentiviral transduction at a multiplicity of infection (MOI) of 5, following an established “spinfection” protocol [18]. Specifically, cells exposed to viral suspension containing 10 μg/mL polybrene (HY-112735, MCE, Princeton, NJ, USA) were centrifuged at 3000× *g* for 1 h at 32 °C. Following centrifugation, cells were incubated at 37 °C and refreshed after 16 h.

### 2.6. Generation of Monoclonal Cell Lines

The immortalized giant panda testicular cells at passage 4 were collected and resuspended with the complete medium. The dilution of the cell suspension was limited to a final concentration of 10–100 cells/mL. Then, 100 μL of the diluted cell suspension was seeded into each well of a 96-well plate, ensuring that over half of the wells remained cell-free to maximize the probability of single-cell distribution in each well. Cell growth was monitored daily under a microscope, and wells containing a single cell were marked to facilitate tracking. Upon proliferation to approximately 80% of confluency, the monoclonal cells were dissociated with 0.25% trypsin-EDTA (Hyclone, Logan, UT, USA) and transferred to 24-well plates for further expansion.

### 2.7. Immunofluorescence

Cells propagated in 96- or 48-well plates were fixed with 4% paraformaldehyde (PFA) for 10 min and permeabilized with 0.25% Triton X-100 for 15 min at room temperature. Subsequently, non-specific binding was blocked by incubating the cells with either blocking buffer (37580, Thermo Fisher Scientific, Waltham, MA, USA) or 3% bovine serum albumin (BSA; 08R81068-CF, MP Biomedicals, Santa Ana, CA, USA) for 2 h at room temperature. After blocking, the cells were incubated with primary antibodies overnight at 4 °C. The primary antibodies utilized were rabbit anti-ACTA2 (1:200; 14395-1-AP, MP Biomedicals, Santa Ana, CA, USA), rabbit anti-SOX9 (1:500; ab185966, Abcam, Cambridge, UK), rabbit anti-SV40 (1:200; 15729S, Cell Signaling Technology, Danvers, MA, USA), rabbit anti-α-tubulin (1:200; 11224-1-AP, MP Biomedicals, Santa Ana, CA, USA), and TRITC-conjugated phalloidin (1:200; 4073ES75, Yeasen Biotechnology, Shanghai, China). For negative controls, the primary antibody was replaced with the isotype rabbit immunoglobulin G. On the following day, after thorough washing, the cells were incubated with donkey anti-rabbit secondary antibody conjugated to Alexa Fluor 488 or 594 (1:400; Yeasen Biotechnology, Shanghai, China) for 2 h at room temperature. After additional washing, cell nuclei were counterstained with DAPI (1:1000; SL7100, Coolaber, Beijing, China) for 5 min at room temperature. Fluorescence images were taken using a Nikon Eclipse 80i fluorescence microscope. To quantify the positive cells, three independent experiments were performed (*n* = 3), with 200 cells analyzed per group in each experiment.

### 2.8. Western Blot Analysis

Total proteins were extracted from the cells using RIPA lysis buffer (DY50002, DeeYeebio, Shanghai, China), followed by centrifugation at 1200 rpm for 10 min. Protein concentrations were determined using a BCA Protein Assay Kit (DY30204, DeeYeebio, Shanghai, China). Later, proteins were separated by SDS-PAGE, transferred to PVDF membranes, and blocked, followed by primary antibody incubation overnight at 4 °C. The primary antibodies used for immunoblotting were rabbit anti-SV40 (1:1000; 15729S, Cell Signaling Technology, Danvers, MA, USA), rabbit anti-ACTA2 (1:200; 14395-1-AP, MP Biomedicals, Santa Ana, CA, USA), and rabbit anti-α-tubulin (1:200; 11224-1-AP, MP Biomedicals, Santa Ana, CA, USA). On the following day, after thorough washing, the blots were incubated with the secondary antibody HRP-conjugated goat anti-rabbit IgG (1:2000; DY60202, DeeYeebio, Shanghai, China). After additional washing, the blots were visualized using a Western Bright ECL Kit (DY30208, DeeYeebio, Shanghai, China) and imaged with a Chemi-Doc XRS system (Bio-Rad, Hercules, CA, USA) via chemiluminescence detection.

### 2.9. RT-PCR Analysis

Total RNAs were extracted from the cells using the Trizol reagent (9109, TAKARA, Tokyo, Japan). Following genomic DNA (gDNA) removal by DNase treatment, complementary DNAs (cDNAs) were synthesized using a cDNA synthesis kit (DY90104, DeeYeebio, Shanghai, China). PCRs were then performed using the synthesized cDNAs as templates. The amplified products were separated by electrophoresis on a 2% agarose gel, stained with nucleic acid dye (DY10214, DeeYeebio, Shanghai, China), and visualized under ultraviolet light. Negative controls entailed total RNAs without reverse transcription (-RT) that were subjected to PCR. The primer sequences and expected RT-PCR product sizes are listed in Table 1.

### 2.10. Functional Validation by Quantitative Real-Time PCR (qPCR)

Functional validation of giant panda TPTCs was performed as previously described [10]. Briefly, the immortalized monoclonal TPTCs at passage 25 were exposed to a serum-free medium harboring ATP (1 mM; Y38247, Shanghai Yuanye Bio-Technology, Shanghai, China), FSK (10 µM; F70001, Psaitong, Beijing, China), or 0.1% ethanol (control) for 24 h. Then, total RNAs were extracted and reversely transcribed using a cDNA synthesis kit (DY90104, DeeYeebio, Shanghai, China). The resultant cDNAs underwent a 30-fold dilution, followed by qPCR using the ChamA Universal SYBR qPCR Master Mix (Q711-02, Vazyme, Nanjing, China) and running on an FQD-96A platform (Bioer, Hangzhou, China). The thermal cycling conditions were 95 °C for 5 min, 40 cycles of 98 °C for 10 s and 60 °C for 30 s, and a final extension at 72 °C for 10 min. *GAPDH* was used as the reference gene, and the relative gene expression level was calculated using the 2^−ΔΔCT^ method. The results were normalized to those in the control. The primer sequences and qPCR product sizes are listed in Table 1.

### 2.11. Transfection

For transfection, the immortalized monoclonal TPTCs at passage 25 were seeded into 24-well plates, and when reaching 50–70% of confluency, they were transfected with GFP plasmids (pGreenPuro; System Biosciences, Palo Alto, CA, USA) using the Hieff Trans™ Liposomal Transfection Reagent (40802ES, Yeasen Biotechnology, Shanghai, China), following the manufacturer’s instructions. Cells were refreshed and fluorescence images were taken 24 and 48 h post-transfection. To quantify the transfection efficiency, three independent experiments were performed (*n* = 3), with 200 cells analyzed in each experiment.

### 2.12. Karyotyping

Karyotyping was carried out on both primary giant panda testicular cells (passages 3–5) and the immortalized monoclonal TPTCs (passage 26). Briefly, cells at roughly 70% of confluency were treated with 0.4 μg/mL colchicine (ST1173, Beyotime Biotechnology, Shanghai, China) for 4 h, swollen with 75 mmol/L KCl, and fixed with methanol–glacial acetic acid (3: 1). The cell suspension was then dropped onto cold slides. After air drying, the slides were stained with DAPI (1:1000; SL7100, Coolaber, Beijing, China) and visualized using a Nikon Eclipse 80i fluorescence microscope. Three independent experiments were performed (*n* = 3), with 100 cells analyzed per group in each experiment.

### 2.13. DNA Content Analysis

A DNA content analysis was conducted on both primary giant panda testicular cells (passages 3–5) and the immortalized monoclonal TPTCs (passage 28). The DNA content was analyzed by flow cytometry as previously described [19], using a FACS analyzer (BD Biosciences, San Jose, CA, USA) and ModFit LT 32 software version 4.0 (Verity Software House, Topsham, Maine, USA).

### 2.14. Statistics

Unless otherwise specified, all data are presented as the mean ± standard error of the mean (SEM) from three independent experiments. The statistical significance between the two groups was assessed using a two-tailed Student’s *t*-test. Differences were considered statistically significant at *p* < 0.05 (*) and highly significant at *p* < 0.01 (**).

## 3. Results

### 3.1. Immortalization of Giant Panda Testicular Somatic Cells

**Figure 1:**
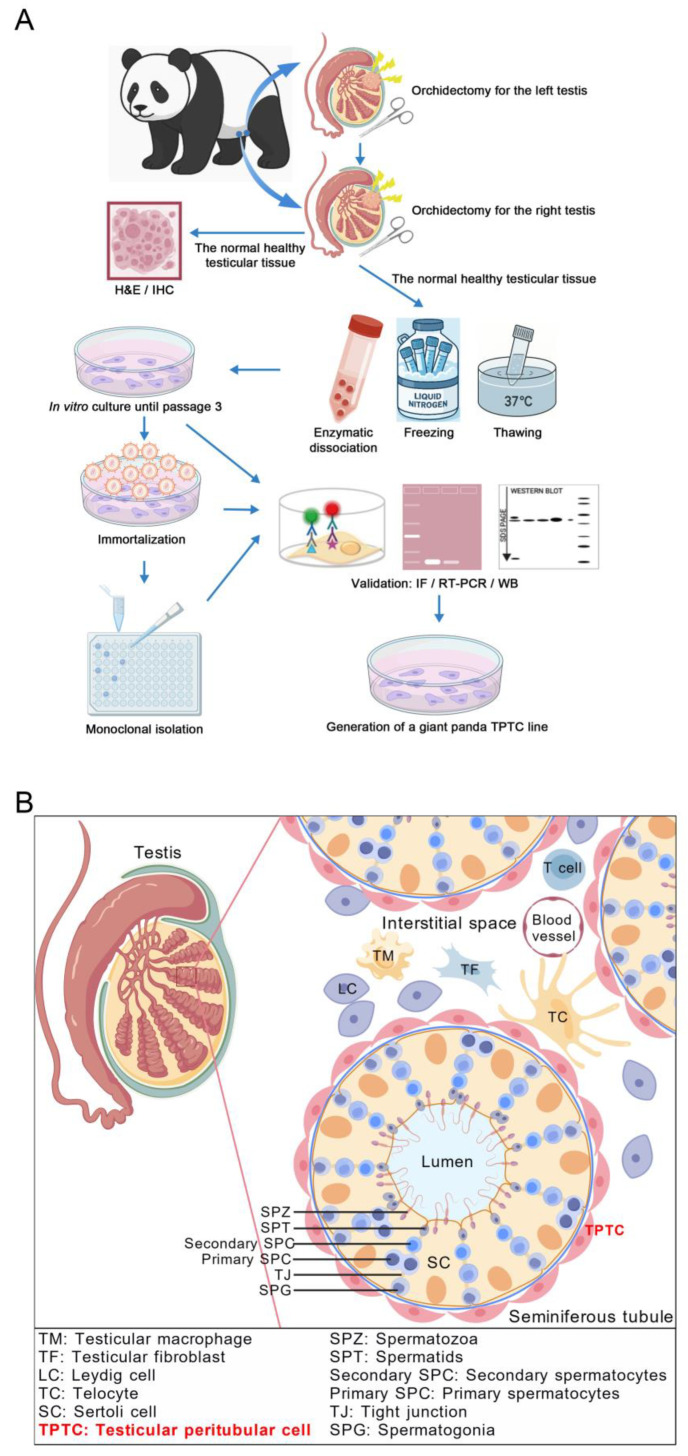
(**A**) Workflow of generation of immortalized TPTC line from giant pandas and (**B**) schematic overview of seminiferous tubule cross-section and testicular interstitial tissue, with TPTCs highlighted in red.

**Figure 2:**
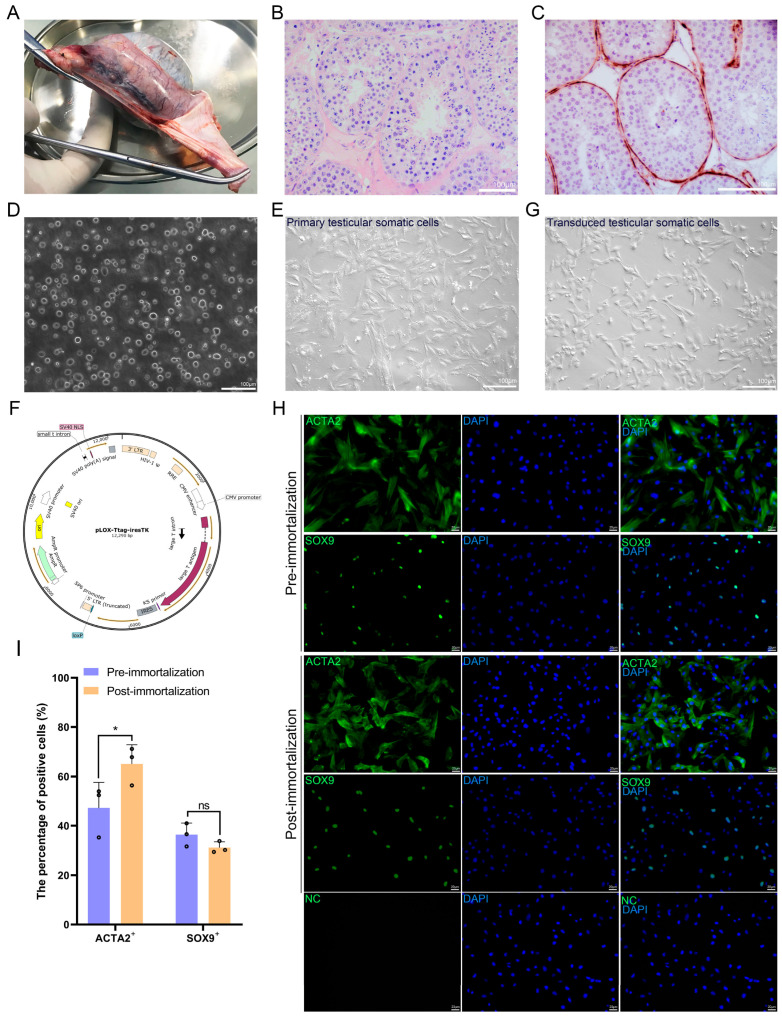
Immortalization of giant panda testicular somatic cells. (**A**) Image of right testis removed from adult giant panda. (**B**) H&E staining of giant panda testis section. Bar = 100 μm. (**C**) Immunostaining for ACTA2 on giant panda testis section. Bar = 100 μm. (**D**) Image of dissociated single cells from giant panda testis. Bar = 100 μm. (**E**) Image of primary testicular somatic cells prior to lentiviral transduction. Bar = 100 μm. (**F**) Map of transfer vector plox-Ttag-iresTK. (**G**) Image of transduced testicular somatic cells. Bar = 100 μm. (**H**,**I**) (**H**) Immunofluorescence images and (**I**) quantification of ACTA2^+^ and SOX9^+^ cells in primary and immortalized testicular cell populations. Bar = 20 μm. Data are presented as mean ± SEM of three independent experiments (*n* = 3). *: *p* < 0.05. ns: Non-Significant. Open circles (o) represent individual data points.

The aim of this study was to generate an immortalized monoclonal cell line with TPTC identities from giant pandas (Figure 1A,B). First, we obtained the right testis from an adult giant panda (Figure 2A), who had testicular tumors in both testes and underwent bilateral orchidectomy. Upon removal of the right testis, the tumorous tissue was carefully separated, and only the normal healthy tissue was utilized for a battery of subsequent experiments. H&E staining revealed the normal histology of the collected healthy testicular tissue (Figure 2B), and immunostaining for ACTA2, a conserved marker also used for giant panda TPTCs [16], showed the localization of TPTCs to the exterior of seminiferous tubules and one single layer of TPTCs surrounding each tubule (Figure 2C).

Next, we conducted mechanical dissection and enzymatic dissociation for the testicular tissue and obtained a single-cell suspension (Figure 2D). The single-cell suspension was then aliquoted and cryopreserved. Later, an aliquot of cells was thawed and subjected to the standard culture until passage 3, when no evident germ cells were observed in the culture (Figure 2E). To induce immortalization, we delivered the plox-Ttag-iresTK vector encoding SV40 large T antigen (Figure 2F) to these cells by utilizing an optimized lentiviral transduction protocol. The transduced cells displayed no manifest morphological alteration (Figure 2G). Immunostaining for ACTA2 and SOX9 (which characterizes Sertoli cells) revealed the presence of TPTCs and Sertoli cells in both the primary and immortalized testicular cell populations (Figure 2H,I).

### 3.2. Monoclonal Isolation and Expansion

**Figure 3:**
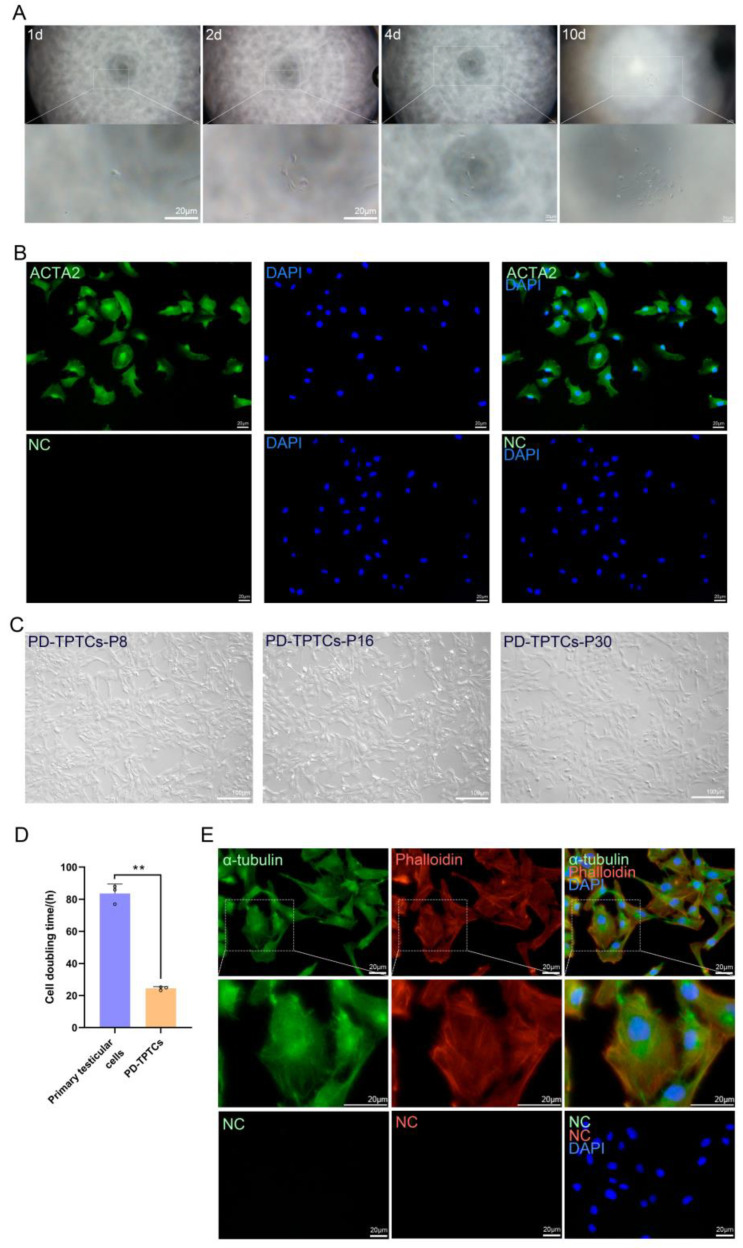
Monoclonal isolation and expansion. (**A**) Images of cell clones in 96-well plates on days 1, 2, 4, and 10 after seeding. Bar = 100 or 20 μm. (**B**) Immunofluorescence images of all-ACTA2^+^-cell clone. Bar = 20 μm. (**C**) Images of monoclonal cell line (PD-TPTCs) at passages 8, 16, and 30. Bar = 100 μm. (**D**) Cell doubling time of primary testicular somatic cells and PD-TPTCs. Data are presented as mean ± SEM of three independent experiments (*n* = 3). **: *p* < 0.01. Open circles (o) represent individual data points. (**E**) Co-staining for α-tubulin and phalloidin on PD-TPTCs. Bar = 20 μm.

As the obtained immortalized cells harbored testicular somatic cell types other than TPTCs, we next performed monoclonal isolation of the immortalized testicular cells at passage 4 by way of limiting dilution. Approximately 10 days after seeding, cell clones were discerned in 96-well plates (Figure 3A). Overall, seven cell clones were obtained, and by ACTA2 immunostaining, we found that only one clone was all positive for ACTA2 staining (Figure 3B), while the others were just partially positive for ACTA2 staining. The only all-ACTA2^+^-cell clone was, hence, expanded and designated as PD-TPTCs. These cells consistently displayed a spindle or elongated fibroblast-like shape with a relatively compact arrangement and showed no evident morphological alteration during cultivation (Figure 3C). In comparison with the primary testicular somatic cells, PD-TPTCs showed substantially decreased cell doubling time (Figure 3D), suggestive of accelerated cell proliferation by immortalization. To assess the cytoskeletal integrity, we conducted co-staining for α-tubulin and phalloidin on PD-TPTCs at passage 25 and found a normal cytoskeletal organization without multinucleation (Figure 3E). This cell line has been cultured for over five months and passaged more than 50 times without apparent morphological changes to date, demonstrating robust proliferative capacity and morphological stability during long-term culture.

### 3.3. Characterization of PD-TPTCs

**Figure 4:**
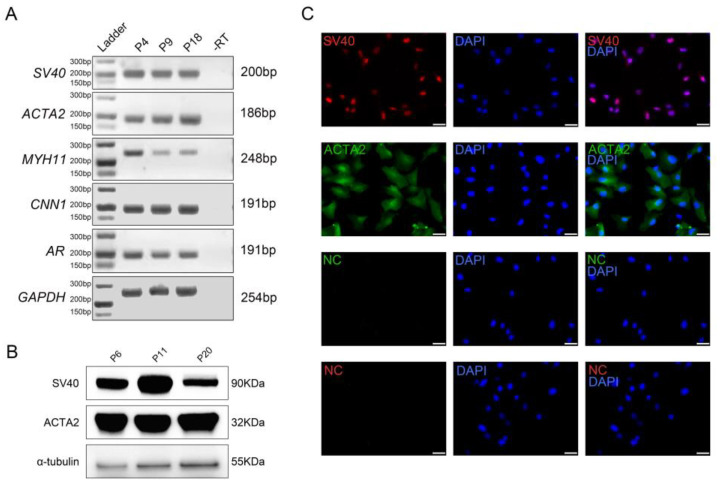
Characterization of PD-TPTCs. (**A**) RT-PCR analysis showing expression of *SV40*, *ACTA2*, *MYH11*, *CNN1*, and *AR* in different passages of PD-TPTCs at mRNA level. *GAPDH* was used as internal control. -RT indicates negative control without reverse transcription. (**B**) Immunoblotting of SV40 and ACTA2 in different passages of PD-TPTCs. α-tubulin served as loading control. (**C**) Immunofluorescence staining of SV40 and ACTA2 on PD-TPTCs at passage 21. Bar = 20 μm.

To characterize the generated monoclonal cell line (PD-TPTCs), we detected the expression of classical TPTC markers by using RT-PCR, Western blot, and immunofluorescence. The RT-PCR analysis revealed the presence of transcripts for canonical TPTC markers, i.e., *ACTA2*, *MYH11*, *CNN1*, and *AR*, in different passages of the PD-TPTC line (Figure 4A), indicative of their TPTC identity. The *SV40* transcript was also detected, demonstrating the successful immortalization of PD-TPTCs (Figure 4A). Consistently, the Western blot (Figure 4B) and immunofluorescence analyses (Figure 4C) showed the expression of SV40 and ACTA2 at the protein level. Overall, these results demonstrate that the generated monoclonal cell line retains key phenotypic features of TPTCs.

### 3.4. Functional Validation of PD-TPTCs

**Figure 5:**
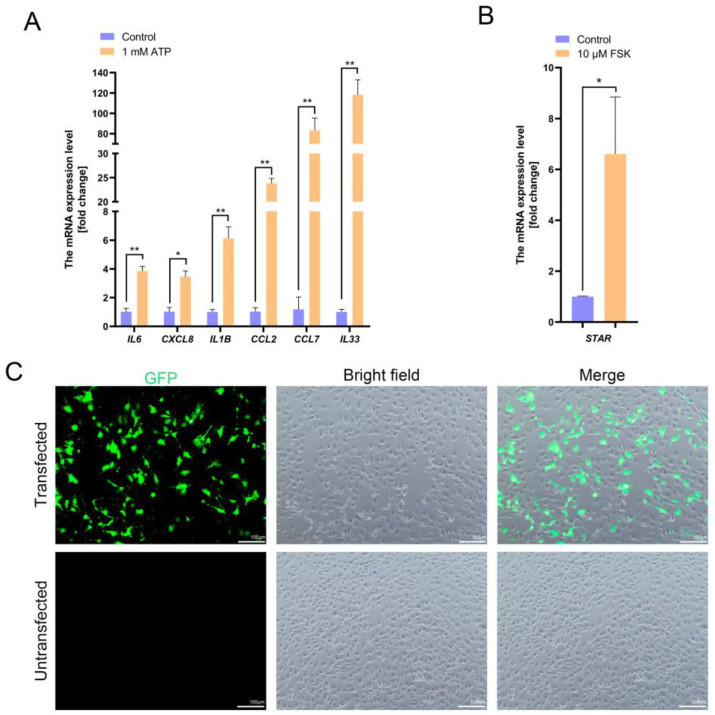
Functional validation of PD-TPTCs. (**A**,**B**) qPCR analysis of (**A**) inflammation-related genes and (**B**) *STAR* in PD-TPTCs in response to (**A**) ATP or (**B**) FSK treatment. Data are presented as mean ± SEM of three independent experiments (*n* = 3). *: *p* < 0.05, **: *p* < 0.01. (**C**) Images of PD-TPTCs with or without transfection under fluorescent and/or bright field. Bar = 100 μm.

To functionally validate the generated monoclonal cell line (PD-TPTCs), we tested its response to inflammatory or steroidogenic stimuli. As expected, treatment of PD-TPTCs (passage 25) with 1 mM ATP for 24 h significantly increased the mRNA level of inflammation-related genes such as *IL6*, *CXCL8*, *IL1B*, *CCL2*, *CCL7*, and *IL33* (Figure 5A), indicative of the conserved pro-inflammatory response. Stimulation of PD-TPTCs (passage 25) with 10 μM FSK for 24 h also led to the elevated *STAR* mRNA level (Figure 5B), suggesting the steroidogenic response that is a hallmark of TPTC functions. In addition, to assess whether the generated PD-TPTC line is suitable for mechanistic studies, we measured its transfection efficiency by introducing GFP plasmids using lipofection. After 48 h, we found that 54.39 ± 4.49% of PD-TPTCs (passage 25) showed green fluorescence (*n* = 3, Figure 5C), suggesting that they are amenable to transfection. These results thus demonstrate that the generated monoclonal cell line retains key functional characteristics of TPTCs, including inflammatory signaling, steroidogenic potential, and transfection competency.

### 3.5. Karyotyping of PD-TPTCs

**Figure 6:**
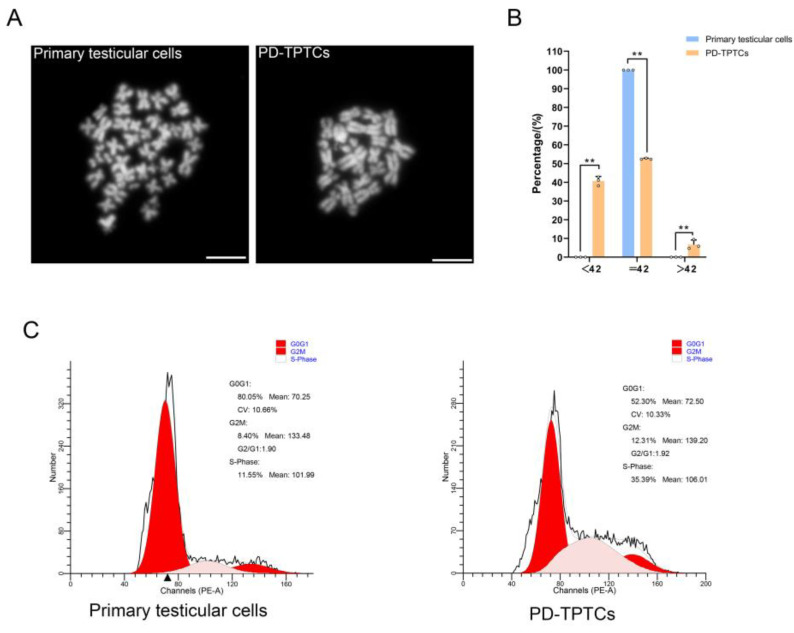
Karyotyping of PD-TPTCs. (**A**) Images of metaphase chromosomal spreads of primary testicular cells and PD-TPTCs. Bar = 5 μm. (**B**) Percentages of cells with normal (42 chromosomes) or abnormal karyotypes (≠42 chromosomes) in primary testicular cells and PD-TPTCs. Data are presented as mean ± SEM of three independent experiments (*n* = 3). **: *p* < 0.01. Open circles (o) represent individual data points. (**C**) DNA contents of primary testicular cells and PD-TPTCs.

Finally, to investigate whether SV40 large T antigen-mediated immortalization induces chromosomal anomalies in the generated monoclonal cell line, we conducted karyotyping and DNA content analyses on both primary testicular cells and PD-TPTCs at passages greater than 25. The karyotyping analysis disclosed karyotypic anomalies in the PD-TPTC line. To be more specific, while the primary testicular cells consisted of 21 pairs of chromosomes characteristic of the giant panda, a considerable number of PD-TPTCs exhibited chromosomal loss (Figure 6A,B). Consistently, the DNA content analysis uncovered a greater proportion of cells in the S- or G_2_/M-phase in PD-TPTCs than in the primary testicular cells (Figure 6C), suggestive of the altered DNA ploidy and potential genomic instability.

## 4. Discussion

TPTCs, a major somatic cell type in the testis, were first observed through electron microscopy in the mid-20th century and described as contractile cells arranged around the seminiferous tubules that exhibit the morphological characteristics of smooth muscle cells [20]. In our previous study, cells located in the giant panda peritubular compartment were found to express ACTA2 [16], suggesting the presence of TPTCs with contractile features in this species as well. For a long time, their role was simply perceived as purely mechanical, assisting in the transport of sperm towards the epididymis. Yet, recent studies have revealed that TPTCs possess a wide array of functions beyond mechanical support, including immune modulation, maintenance of the testicular niche, ECM remodeling, and SSC fate determination [1]. In mice, TPTCs have been demonstrated to secrete essential regulatory factors such as GDNF and CSF1 that influence the SSC behavior [21], and by conditional knockout using TPTC-specific Cre drivers, their indispensable roles in spermatogenesis have been studied in detail [22]. In humans, TPTCs can be isolated and cultivated in vitro [9], and the primary TPTCs display the classic smooth muscle-like phenotype, express markers such as ACTA2, CNN1, and CALD1, and are capable of secreting key factors like GDNF, IL-6, and CXCL8 that are important to SSC maintenance and immune homeostasis [11]; thus, they have been extensively harnessed to investigate the effects of various compounds and hormones (e.g., FSK and ATP) on TPTC functions. Nonetheless, the primary culture of TPTCs is associated with issues such as replicative senescence and donor-derived variability. In non-human primates, an immortalized TPTC line from the common marmoset (*Callithrix jacchus*) has been successfully established through delivering hTERT into the cells. This immortalized cell line closely resembles its human counterpart in terms of marker expression and functional response [10], making it one of the most physiologically relevant TPTC models suitable for comparative studies.

While there are studies on TPTCs in mice, humans, and non-human primates that have significantly advanced the understanding of their functions, corresponding research in specific endangered species such as giant pandas remains extremely limited, which is largely ascribed to the paucity of samples and the lack of robust in vitro cell models. Indeed, TPTCs tend to undergo replicative senescence under in vitro conditions, gradually losing proliferative capacity with time [5]. In this study we have, for the first time to the best of our knowledge, established an immortalized monoclonal cell line with TPTC identities from giant pandas (PD-TPTCs). The generated cell line was demonstrated to express key TPTC markers such as ACTA2, MYH11, CNN1, and AR. Further functional assays revealed that they could respond to ATP stimulation with a pro-inflammatory gene expression profile and to FSK with increased steroidogenic activity. Moreover, they were shown to be amenable to lipofection. Hence, the established PD-TPTC line represents a promising cellular model to investigate the mechanisms underlying the testicular niche, spermatogenesis, and reproductive disorders in giant pandas.

Indeed, testis samples from giant pandas are extremely rare. Typically, they are only available postmortem, and in some exceptional cases, surgically excised tissue can be collected during medical interventions. In this study, testicular tumors were, fortunately, identified at an early stage, and the testis was only partially compromised, with three clearly different sections, i.e., normal tissue, tumorous tissue, and necrotic tissue, macroscopically discerned. Notably, all testicular tissue used in this study was taken from the central portion of macroscopically normal tissue located away from the lesion margin, and H&E staining revealed the normal histology of the collected healthy tissue, showing the typical seminiferous tubule architecture and the harbored various stages of germ cells. Moreover, the karyotyping analysis disclosed the normal karyotype of primary testicular cells that consisted of 21 pairs of chromosomes characteristic of the giant panda, together minimizing the possibility of contamination with transformed cells. Despite all these, we acknowledge the limited representativeness of the findings, since the cell line was derived from a single giant panda testis, which is chiefly due to the paucity of testis samples from this endangered species. In this sense, future studies are needed to corroborate the utility and physiological relevance of the generated cell line.

SV40 large T antigen is a well-acknowledged potent exogenous agent needed to attain cell immortalization [23]. By binding to the tumor suppressor p53 and the retinoblastoma susceptibility protein Rb and inhibiting their functions, SV40 large T antigen enables cells to evade apoptosis and sustain continuous proliferation [24]. Given the lack of approaches to efficiently enriching TPTCs at the moment, we first introduced SV40 large T antigen into primary testicular somatic cells derived from giant pandas by employing an optimized lentiviral transduction strategy termed “spinfection” [18], followed by monoclonal isolation, leading to the ultimate generation of an immortalized monoclonal cell line with TPTC identities (PD-TPTCs). This cell line exhibited robust proliferative capacity and propagated in vitro for over five months, thus providing a reliable cellular resource for downstream functional assays. Notably, no manifest morphological alterations were observed throughout the culture process, and the cells consistently showed the spindle or elongated fibroblast-like morphology with relatively compact arrangement. Nevertheless, karyotyping and DNA content assays revealed that the PD-TPTC line exhibited chromosomal structural alterations or aneuploidy, which is not surprising, as SV40 large T antigen-mediated cell immortalization is typically associated with cell transformation characterized by chromosomal structural or numerical anomalies [25].

ACTA2 has extensively been utilized as a reliable marker for characterization of TPTCs. Recently, by conducting ACTA2 immunostaining on testis sections from an adult giant panda, we characterized the giant panda TPTCs in situ, revealing one single layer of TPTCs surrounding the seminiferous tubules in giant pandas [16] and suggesting that these cells may also exhibit smooth muscle-like phenotypes in this endangered species. Here, to characterize the generated cell line, we performed RT-PCR, immunofluorescence, and Western blot analyses, and the results showed that the PD-TPTC line expressed ACTA2, MYH11, CNN1, and AR. This marker expression pattern highly resembles that in human TPTCs [9], confirming that the generated cell line retains the TPTC characteristics. Among these markers, ACTA2 and CNN1 are key components of the contractile apparatus [11], and their expression suggests that the PD-TPTC line retains the contractile property that is characteristic of myoid cells. CNN1 has also been widely acknowledged as a TPTC marker in non-human primates. It plays a critical role in the fine-tuning of smooth muscle contractility, with its expression level commonly used to assess the functional state of TPTCs [26]. The expression of CNN1 in the generated cell line lends further support to its utility as a reliable marker for TPTCs in giant pandas. In addition, AR was shown to be expressed in the PD-TPTC line, suggesting that these cells may be capable of responding to androgen signaling [27] and, therefore, performing a potential role in testicular paracrine and endocrine regulation.

To functionally validate the generated PD-TPTC line, we applied a previously depicted strategy by Schmid and coauthors. As they reported, treatment of immortalized monkey TPTCs with 1 mM ATP markedly upregulated inflammatory mediators such as IL1B, CCL2, and CCL7, indicative of immune response to local inflammatory stimuli [10]. ATP, as an exogenous purinergic signaling molecule, is massively released upon tissue injury or under stress conditions, acting as a “danger-associated molecular pattern (DAMP)” through the activation of P2X/P2Y receptors and mediating intracellular Ca^2+^ signaling, leading to activation of the NF-κB pathway and the inflammatory factor cascade [28,29]. Here, treatment of PD-TPTCs with 1 mM ATP for 24 h consistently resulted in significant upregulation of inflammatory factors such as IL6, CXCL8, IL1B, CCL2, CCL7, and IL33, indicating a robust response to inflammatory cues, which highly resembles that in immortalized monkey TPTCs, suggesting that the PD-TPTC line generated in this study retains the core biological features of TPTCs that are related to inflammation regulation. In addition, FSK, a well-known adenylyl cyclase activator, is commonly used to stimulate steroidogenic activity by activating the cAMP/PKA signaling pathway and inducing the expression of steroidogenesis-related genes [30,31]. It has been reported that FSK stimulation elevated the expression of STAR in immortalized monkey TPTCs [10], suggesting a supportive role of TPTCs in local steroid homeostasis in testes. Consistently, our results showed that treatment of PD-TPTCs with 1 mM FSK for 24 h also significantly increased the *STAR* mRNA level, pinpointing the steroidogenic response of the generated PD-TPTC line.

To date, no direct experimental evidence has linked TPTC dysfunction to fertility impairment in giant pandas. Despite this, analogous findings from other species suggest that TPTC abnormalities may have a detrimental influence on reproductive health in this endangered species as well. In humans and mice, TPTC dysfunction has been associated with male infertility, testicular dysgenesis, and testicular fibrosis, all hallmarked by disorganized TPTC architecture, dysregulated cytokine secretion, and increased ECM deposition, collectively undermining the SSC niche, impeding SSC maintenance, and reducing sperm output [7,32].

In addition, there is currently no direct evidence that TPTC disfunction causes fertilization defects. But on the other hand, TPTCs’ critical roles in orchestrating the SSC niche and sperm quality represent a vital avenue for future research. With the advent of single-cell omics, TPTC-Sertoli cell co-culture systems, and in vitro organ-on-chip testicular models, future studies are expected to elucidate how TPTCs influence SSC fate, sperm maturation, and ultimate fertilization competence, thereby providing potential strategies to support reproductive preservation in endangered species such as giant pandas.

## 5. Conclusions

In this study, by introducing SV40 large T antigen into giant panda primary testicular somatic cells via lentiviral transduction and the subsequent monoclonal isolation through limiting dilution, we successfully generated an immortalized monoclonal cell line with TPTC identities from giant pandas (PD-TPTCs). This cell line exhibited unlimited proliferative capacity, and it has been cultured for over five months and passaged more than 50 times to date. The PD-TPTC line also expressed key TPTC markers such as ACTA2, MYH11, CNN1, and AR. Additionally, they could respond to ATP and FSK stimulation with a pro-inflammatory gene expression profile and increased steroidogenic activity, and were amenable to lipofection. The generated PD-TPTC line represents a promising cellular model that can be used to investigate the mechanisms underlying the testicular niche, spermatogenesis, and reproductive disorders in giant pandas, and lays the foundation for the development of novel ART and preservation strategies in this endangered species.

## Figures and Tables

**Table 1:** Primer and (q)RT-PCR product information.

Gene	Primer Sequence (5′-3′)	Product Size (bp)	Accession Number
*SV40*	F: TTTGGAGGCTTCTGGGATGC R: CAGTAGCAATCAACCCACACAAG	200	
*ACTA2*	F: TCACCAACTGGGACGACAT R: CCTGGATAGCCACATACATAGC	186	XM_011218625.3
*MYH11*	F: GCAATGCCAAAACGGTCA R: GGAGAGGAATGTGTAGTTGTTGAAG	248	XM_034670322.1, XM_034670323.1, XM_034670324.1, XM_034670325.1
*CNN1*	F: CCCCACGACATTTTTGAGGC R: CCGATGATGTTCCGCCCTTC	191	XM_002930829.4
*AR*	F: ATCCTCACACCCGCATCAAG R: CTGGCCTTCTTCTGCCGTAA	191	XM_002929171.4
*IL6*	F: ACGGATGCTTCCAATCTGGG R: TTGCTTCAGCCACTTGTCCT	269	NM_001304922.1
*CXCL8*	F: CAATGGAGAAGAGGTGTGCCT R: CCAGACCCACACGGAACAAG	192	XM_002928494.4
*IL1B*	F: AGTGCTGCTTCCAAGACCTGAA R: AGCCACAACGACTGACACGAA	116	XM_002926330.4
*CCL2*	F: AGCAGCAAGTGTCCCAAAGA R: TGGCTTTGCAGTTTGCGTTT	138	XM_002912354.4
*CCL7*	F: ACTTCTCTGCCTGCTGCTTAC R: GCTGCTGGTGATTCTTCTGTAG	154	XM_002912355.4
*IL33*	F: AACAGGTGATGGCGTTGATGG R: CCTATGAAGGCGGAAGAAGACC	157	XM_011217254.3, XM_034663833.1, XM_019793249.2
*STAR*	F: GCCACACGCTTCGGAGAGAT R: GTCCACCTGGGTCTGGGATAAG	192	XM_002917230.4
*GAPDH*	F: GTGATGCTGGTGCTGAGTATGT R: CTCCACGATGCCGAAGTTG	254	NM_001304846.1

## Data Availability

All data are included in the article.
